# The Emerging Role of the Salt Tolerance-Related Protein in the Abiotic Stress Response of *Arabidopsis thaliana*

**DOI:** 10.3390/plants14192954

**Published:** 2025-09-23

**Authors:** Anna Fiorillo, Michela Manai, Elisa Falliti, Sabina Visconti, Lorenzo Camoni

**Affiliations:** Cellular and Molecular Biology, Department of Biology, University of Rome Tor Vergata, 00133 Rome, Italy; anna.fiorillo@uniroma2.it (A.F.); michela.manai@uniroma2.it (M.M.); elisa.falliti@alumni.uniroma2.eu (E.F.); visconti@uniroma2.it (S.V.)

**Keywords:** STRP, salt stress, cold stress, stress tolerance, intrinsically disordered proteins, oxidative stress, post-translational modifications, protein ubiquitination, N-terminal-acetylation, abscisic acid

## Abstract

Abiotic stresses severely impair plant growth and productivity. To counteract stress, plants have evolved intricate strategies, including the induction of stress-responsive proteins. The *Arabidopsis thaliana* Salt Tolerance-Related Protein (STRP) has recently emerged as a key player in abiotic stress tolerance. STRP is a small, hydrophilic, intrinsically disordered protein that exhibits the potential to adopt distinct conformations depending on the cellular context. STRP is localized in the cytosol and nucleus and is associated with the plasma membrane. Stress induces the subcellular redistribution of STRP, accompanied by a significant increase (up to ten-fold) in its levels due to reduced degradation by the 26S proteasome. Reverse genetics studies have demonstrated that STRP can mitigate the detrimental effects of oxidative stress and participate in modulating stress-related gene expression. Although the exact mechanism of STRP remains unclear, its physicochemical properties suggest a dual role as a molecular shield, interacting with macromolecules without a fixed conformation, and as a binder of specific defense-related client proteins, adopting a defined tertiary structure. This review provides a comprehensive overview of STRP and its emerging role as a multifunctional player in abiotic stress responses, also highlighting its potential for strengthening crop resilience and maintaining agricultural productivity under global climate challenges.

## 1. Introduction

Abiotic stresses, such as salinity, drought, and extreme temperature, are among the most significant factors limiting plant growth and agricultural productivity globally [1,2,3]. As sessile organisms, plants are continuously exposed to fluctuating environmental conditions that can negatively impact their metabolism, development, and reproductive success [4,5,6]. Plants exposed to environmental stress undergo a wide range of cellular and molecular alterations. Some of these changes are non-adaptive, reflecting damage such as perturbations in membrane dynamics, protein stability, or enzymatic activity. Others, however, represent adaptive adjustments that enable plants to withstand unfavorable conditions and maintain cellular homeostasis [6]. Among abiotic stresses, cold and salinity impose major constraints on plant cells, leading to membrane destabilization, protein denaturation, and excess accumulation of reactive oxygen species (ROS), which together impair photosynthesis and metabolism while damaging cellular structures [7]. To withstand such conditions, plants have evolved a sophisticated network of physiological and molecular mechanisms, including remodeling of membranes and cell walls, accumulation of compatible solutes to stabilize proteins and maintain osmotic balance, and activation of antioxidant systems to detoxify ROS [6,8,9]. At the molecular level, abiotic stresses reprogram gene expression through both transcriptional and epigenetic mechanisms, leading to the induction of genes encoding stress-induced proteins. These proteins represent a central strategy in plant defense, as they preserve cellular structures, maintain metabolic stability under adverse conditions, and activate specific signaling pathways [10,11,12].

In recent years, the Salt Tolerance-Related Protein (STRP), encoded by the *At1g13930* gene in *Arabidopsis thaliana*, has emerged as a novel stress-related protein involved in mitigating the adverse impact of salt and cold stresses on the plant [13,14,15].

Until a few years ago, the function of STRP had not been characterized. STRP has often been identified in large-scale proteomics studies, where it was reported as an uncharacterized protein, or with names based on presumed sequence similarities with known proteins, such as oleosin-B3-like protein [16], nodulin-related protein 2 [17], or expressed protein with weak similarity to 60S ribosomal subunit [18].

In addition to a series of scattered information, more recently, some studies have started to focus on the role of STRP in the response of *A. thaliana* to abiotic stress, with particular reference to salt stress, hence the name of the protein [19].

## 2. Role in Abiotic Stress Tolerance

STRP was first discovered through a gene mining approach aimed at isolating genes involved in stress tolerance in the halophilic plant *Thellungiella halophila*, a close relative of *A. thaliana* [13]. This approach allowed the identification of the *T. halophila* gene *ST6-66*, which confers salt tolerance to *A. thaliana* when heterologously expressed. In *A. thaliana*, the homolog with the highest similarity (79%) to the *ST6-66* is *At1g13930* [13]. Notably, a T-DNA insertion line of *At1g13930* (SALK_076125) was hypersensitive to salt stress, indicating that this gene plays a significant role in salt tolerance [13].

A significant step toward elucidating the potential mechanism underlying the tolerance conferred by STRP was achieved through a proteomic analysis aimed at identifying proteins involved in the temperature stress response in *A. thaliana* [20]. Among the differentially represented proteins, STRP exhibited the most pronounced increase in response to heat and cold stress [20]. Moreover, the stress-induced accumulation of STRP is not restricted to temperature-related stimuli; a similar upregulation has also been observed under high salinity conditions, confirming that STRP may play a broader role in plant responses to multiple abiotic stresses [15].

A deeper insight into the mechanism underlying protein accumulation revealed that the increase in STRP under cold and salt stress is not due to transcriptional activation, but rather to an increased protein stability [14]. Under physiological conditions, STRP is an unstable protein destined for degradation by the 26S proteasome, while cold and salt stress inhibit its degradation [14,15].

STRP is localized in the cytosol, in the nucleus, and is associated with the plasma membrane [14,21]. Under cold stress, the plasma membrane fraction of STRP decreases, while the cytosolic and nuclear fractions increase [14]. The following sections of this review will clarify how the intracellular redistribution of the protein could be relevant to its function.

The identification of STRP as a potential player involved in tolerance mechanisms to various stresses prompted further studies to achieve a more comprehensive insight into its mechanistic role in abiotic stresses.

A broad understanding of STRP function has primarily relied on the analysis of genetically modified plants, including the *loss-of-function* mutant (SALK_076125) and overexpression lines, which have collectively contributed to dissecting its involvement in stress responses.

Per se, the *STRP* mutation causes some phenotypic alterations even under physiological conditions, as the *strp* mutant shows shorter hypocotyls and reduced rosette leaf area [14]. Although this evidence suggests a role for STRP in growth and development processes, the research mainly focused on its involvement in stress responses.

In particular, cold and salt stress induce excessive ROS accumulation in the *strp* mutant, due to a reduced activity of cellular antioxidant systems, which leads to increased lipid peroxidation and loss of membrane integrity [14,15]. Conversely, STRP overexpression mitigates the salt stress-induced damage, resulting in a hyposensitive phenotype in redox imbalance and chlorophyll depletion induced by excess salinity [15].

Another noteworthy aspect of STRP’s role in stress tolerance is its involvement in abscisic acid (ABA) signaling, pivotal in plant responses to abiotic stresses [22,23]. ABA regulates various stress-related processes, including stomatal closure, seed germination, root development, and the activation of stress-responsive genes [23,24]. STRP is markedly accumulated upon exogenous ABA application [14], which also promotes the transcriptional activation of the *STRP* gene [14,25]. The *strp* mutant exhibits general hyposensitivity to the hormone in various ABA-regulated processes, including stomatal closure, and altered expression of ABA-responsive genes [14]. Moreover, upon cold and salt stress, the expression of *NCED3*, encoding the enzyme that catalyzes the rate-limiting step of ABA biosynthesis, was impaired in the *strp* mutant, resulting in reduced ABA accumulation [14]. These data pinpoint a role for STRP as a positive regulator of ABA signaling and suggest the existence of a circuit where the stress-induced accumulation of STRP can contribute to increasing ABA synthesis, which in turn further promotes STRP increase by transcriptional activation.

## 3. Molecular Features

STRP is a small (16.2 kDa) acidic, highly hydrophilic protein characterized by a predominance of polar residues and only a few short hydrophobic stretches [14]. In *A. thaliana*, a homologous protein known as Nodulin-Related Protein 1 (NRP1), which shares 55% amino acid sequence identity with STRP, has been identified. Although it has been suggested to play a role in responses to heat stress [26], the function of NRP1 is still largely uncharacterized. Similar proteins are broadly conserved across plant species (Supplementary Figure S1) [27], with several members proposed to be involved in stress response mechanisms [13,28].

In addition to the aforementioned analysis of *T. halophila* ST6-66, the characterization of the wheat protein WCI16, which exhibits 42% amino acid sequence identity with STRP, has contributed to a deeper understanding of STRP’s molecular properties [28]. WCI16 is a hyperhydrophilic, largely unstructured protein showing cryoprotectant activity and solubility after boiling. Given that these characteristics are typical of Late Embryogenesis Abundant (LEA) proteins [29], it has been proposed that, despite the absence of sequence similarity, WCI16 is an Intrinsically Disordered Protein (IDP) [28,30,31] representing a member of a new class of LEA proteins [29]. Similarly, STRP lacks canonical motifs typically found in LEA proteins, although it shares some of their general physicochemical properties [14].

A subset of LEA proteins is included within the broader category of hydrophilins, which are defined by specific physicochemical features: a glycine content above 6% and a Grand Average of Hydropathy (GRAVY) value lower than −1 [29,32,33,34]. Although STRP contains 9% glycine, its GRAVY value is −0.88, which does not meet the criteria for classification as hydrophilin. Nevertheless, STRP displays some other properties typical of hydrophilins, including cryoprotective activity and thermal stability [14].

**Figure 1:**
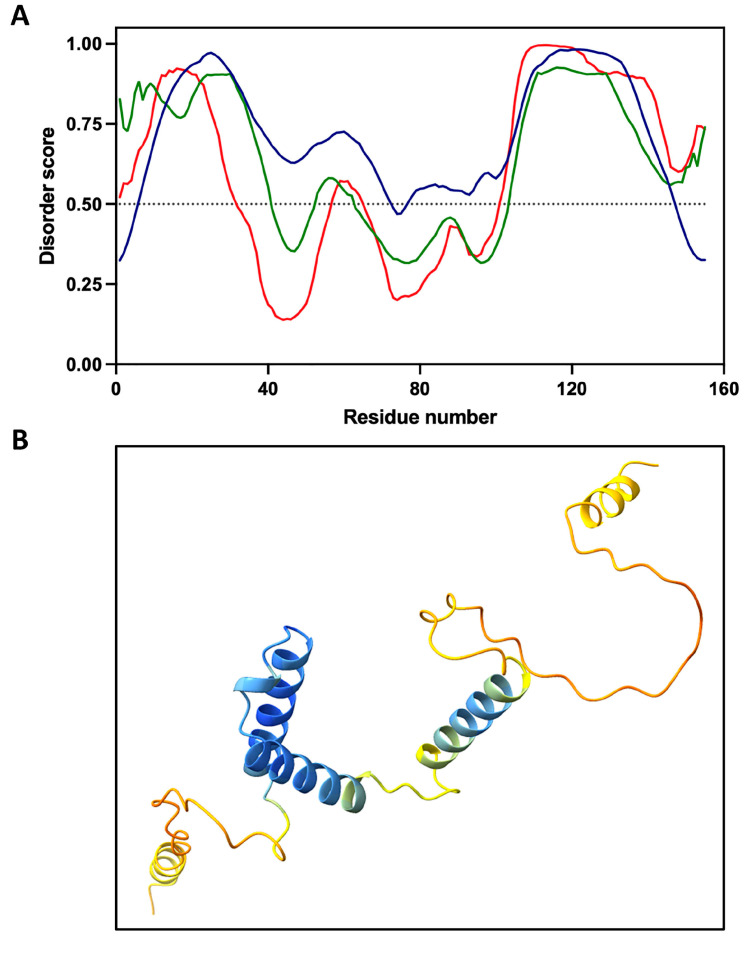
Proposed structural features of STRP. (**A**) Bioinformatic prediction profile of intrinsic disorder along the amino acid sequence of STRP. Blue line, IUPred3 [37]; green line, prDOS [38]; red line, Espritz [39]. (**B**) Prediction of STRP secondary structure with Alphafold 3, with colors indicating different confidence classes [40]. Orange, pLDDT < 50; yellow, 50 < pLDDT < 70; cyan, 70 < pLDDT < 90; blue, pLDDT > 90.

Structural predictions based on bioinformatic analyses indicate that STRP does not adopt a well-defined secondary structure, with only the central region of the polypeptide predicted to acquire a stable conformation (Figure 1A), possibly in a context-dependent manner [14]. The prediction of the STRP structure using the deep learning-based AlphaFold 3 algorithm (Figure 1B) provides more accurate information about the protein structure, assigning different confidence classes to the predicted structure based on pLDDT (Predicted Local Distance Difference Test) values, which is an efficient disorder predictor [35]. STRP shows extensive regions with pLDDT below 50 (orange) and between 50–70 (yellow), indicating that they are unstructured or flexible, respectively. In contrast, the central area has pLDDT values greater than 70%, indicating regions with a probable defined structure (cyan and blue).

The intrinsically disordered nature of STRP is consistent with the anomalous electrophoretic behavior of the protein. Indeed, despite a calculated theoretical molecular mass of 16 kDa, the protein exhibits an apparent molecular mass of approximately 25 kDa [14]. This discrepancy is consistent with the behavior of IDPs, which typically bind less Sodium Dodecyl Sulfate (SDS) and display a reduced electrophoretic mobility during SDS-PAGE [36].

## 4. Regulation of *STRP* Expression

The extensive availability of transcriptomic datasets from microarray and RNA-seq experiments has enabled the generation of a comprehensive overview of *STRP* expression dynamics [41,42,43]. The *STRP* gene (*At1G13930*) displays a relatively ubiquitous and stable expression pattern across tissues, organs, and developmental stages [42,43]. Moreover, although some fluctuations in expression levels are observed, the gene is not markedly regulated in response to abiotic stresses. [43,44]. In Supplementary Figure S2, a graphical overview of the pooled RNA-seq data illustrating *STRP* expression in relation to tissue specificity and responses to abiotic stress conditions is shown [43]. The relatively stable expression profile of STRP further indicates that the elevated protein accumulation under cold and salt stress conditions results predominantly from reduced degradation rather than enhanced transcription.

It is noteworthy that *STRP* can undergo alternative splicing, resulting in three transcript variants (*At1G13930.1*, *At1G13930.2*, and *At1G13930.3*), which encode the same protein but differ in their 5′ and 3′ untranslated regions (UTRs) [16]. Alternative splicing at 5′-UTRs can affect translational efficiency, and mRNA stability and localization, whereas splicing in the 3′-UTR influences mRNA localization, stability, and microRNA or RNA-binding protein interactions [45]. In *A. thaliana*, it is well reported that these mechanisms are involved in fine-tuning post-transcriptional regulation in response to developmental and environmental cues [45,46]. Interestingly, splicing variants *At1G13930.2* and *At1G13930.3* can base-pair with the transcript of the neighboring gene *AT1G13940*, located in the same chromosomal region but arranged in opposite orientation, and therefore transcribed from the opposite DNA strand in the 5′ to 3′ direction [16]. During cold acclimation, pairing leads to the production of a *cis*-natural antisense small interfering RNA (*cis*-nat-siRNA), resulting in post-transcriptional silencing of *AT1G13940*, which encodes a putative T-box transcription factor [47].

Although the mechanisms underlying the generation of the different transcript variants and the functional relevance of *STRP* 5′- and 3′-UTRs’ alternative splicing are still unknown, the involvement of STRP in tolerance mechanisms toward abiotic stress suggests that this process may represent an additional regulatory layer contributing to its protective role.

## 5. Post-Translational Modifications

One of the key aspects that STRP research is currently investigating concerns the potential post-translational modifications involved in regulating its function under stress conditions. It has been shown that proteasome inhibition leads to increased protein levels even in the absence of stress, implying that regulation of protein stability via the ubiquitin proteasome system (UPS) is involved in STRP-mediated stress responses [14,15]. Ubiquitination of target proteins at the ε-amino groups of Lys residues represents a crucial step for their recognition and subsequent degradation by the 26S proteasome. Interestingly, a recent analysis of the ubiquitin-modified proteome demonstrated STRP ubiquitination, revealing the presence of six ubiquitinated Lys in leaves and three in roots of *A. thaliana* [48].

Although no E3 ubiquitin ligase has yet been identified as a direct candidate involved in STRP ubiquitination, it is noteworthy that an F-box protein, the core component of the SCF (SKP1, Cullin, F-box) E3 ligase complex responsible for substrate recognition [49,50], was found to co-purify with STRP within the same macromolecular complex [19] (see Section 6). Although experimental evidence is still lacking, this observation raises the possibility that this F-box may be involved in STRP ubiquitination.

Moreover, quantitative N-terminomics studies demonstrated that STRP is N-terminally acetylated by the N^α^-terminal acetyltransferase B (NatB) complex [51,52]. N-terminal (Nt)-acetylation is a poorly understood post-translational modification that can potentially affect protein folding, localization, protein–protein interactions, and degradation, by modulating protein interaction with the UPS [53,54,55,56]. Remarkably, it is known that NatB impacts diverse development processes and is essential for adaptation to salt and osmotic stress [52,57], suggesting that the Nt-acetylation status of STRP may contribute to regulating its stability and function under stress conditions.

## 6. Subcellular Localization

The subcellular redistribution of STRP in response to stress may be relevant to its function in tolerance mechanisms. STRP may function as a stress sensor at the plasma membrane level, as membrane-associated proteins are known to perceive extracellular cues, such as low temperature, and transduce them into intracellular signaling responses [58,59]. STRP could associate with membrane phospholipids or plasma membrane docking proteins, and its release could be due to cold-triggered alterations of the physicochemical properties of the plasma membrane. Similar mechanisms are common in plants: several stress sensors, such as heat shock proteins (HSPs), G proteins, and phospholipases C and D, are anchored to lipids (e.g., sphingolipids, sterols, and glycosylphosphatidylinositol) or plasma membrane proteins [60,61,62]. Alternatively, the dissociation of STRP from the membrane may occur through an indirect mechanism, representing a downstream consequence of signaling pathways triggered by low-temperature exposure. In this scenario, temperature stress could activate specific mechanisms modulating STRP localization.

The increase in STRP in the cytosol and into the nucleus, observed in response to cold and high salinity stress, is *bona fide* relevant in tolerance mechanisms. Based on the typical properties of IDPs, STRP could stabilize the cytoplasmic environment mainly through non-enzymatic physicochemical mechanisms, preventing protein denaturation or aggregation while preserving the enzymatic activities and the functionality of structural proteins. This mechanism is shared among several plant IDPs, many of which are upregulated under stress conditions [63]. Among these, ERD10 and ERD14, IDPs belonging to group 2 of LEA proteins in *A. thaliana*, are accumulated in response to stresses such as high salinity, drought, and low temperatures and can prevent the aggregation and/or inactivation of stress-labile enzymes [64]. LEA7, another cytosolic IDP, can protect lactate dehydrogenase and other soluble enzymes of *A. thaliana* during freezing and dehydration stress by preventing protein aggregation in the cytosol [65]. Therefore, the molecular shield activity of STRP may also extend to ROS-scavenging enzymes, potentially explaining the redox imbalance observed in the *strp* mutant under stress conditions [14,15].

In the nucleus, the accumulation of STRP is accompanied by increased chromatin binding [14]. Under environmental stress, chromatin remodeling is a crucial step for the remodulation of gene expression and activating appropriate defense responses [66,67]. Therefore, in response to cold stress, STRP may contribute to cellular genetic reprogramming by promoting chromatin remodeling and thus the expression of specific stress-activated genes. Interestingly, STRP was identified as part of the interactome of DEK domain-containing protein 3 (DEK3), a chromatin-associated factor involved in modulating DNA topology and accessibility through its interaction with histones H3 and H4, DNA, and various nuclear proteins [19]. Interactome analysis revealed that DEK3 interacts with 17 protein partners linked to key cellular processes such as growth, development, and stress responses [19]. The identified proteins include the DNA topoisomerase 1α (Top1α) [68], the component of the cohesion complex SCC3 [69], the putative cohesion-associated factor PDS5 [70], Nitrilase1 [71], and a PWWP domain-containing protein [72]. At different levels, these proteins are known to be involved in cytokinesis and the maintenance of genome stability. Other identified proteins are implicated in chromatin dynamics, such as the candidate transcription factor Short Life 1 protein (SHL1) [73], Histone Deacetylase 3 (HDA3/HDT1) [74]. Although the nuclear role of STRP is still largely unknown, the identification of STRP in the nucleus within the DEK3 interactome proposes its involvement in epigenetic processes regulating growth and development, a possibility supported by the phenotypic alterations observed in the *strp* mutant grown in physiological conditions [14].

The interactome also includes an F-box protein (see Section 5), possibly involved in the regulation of the levels of DEK3 or its associated protein, as well as the stress-induced COLD-RESPONSIVE PROTEIN 6.6 (COR6.6/KIN2), which plays a role in abiotic stress adaptation [75]. The presence of both STRP and COR6.6/KIN2 within the DEK3 interactome has prompted preliminary investigations into the functional role of DEK3 in salt stress responses. Experimental data indicate that DEK3 represses the transcriptional response to salt stress by maintaining chromatin in a compacted state, thereby restricting access to stress-activated transcription factors [19]. STRP may exert an antagonistic effect within this regulatory framework, potentially counteracting DEK3-mediated repression and facilitating the activation of stress-inducible genes. Analysis of the STRP interactome and its dynamics in response to stress is necessary to more clearly define the functional relationships between STRP and its targets, including DEK3 and its interactors.

## 7. Mechanism of Action of STRP: Hypothesis and Future Perspectives

In recent years, research on the previously long-uncharacterized STRP has started to reveal its important role in plant tolerance responses to environmental stresses. Although several aspects have been clarified, including structural properties, mechanisms regulating the stress-induced accumulation, and subcellular localization, the precise molecular mechanism underlying STRP’s protective role remains unknown.

**Figure 2:**
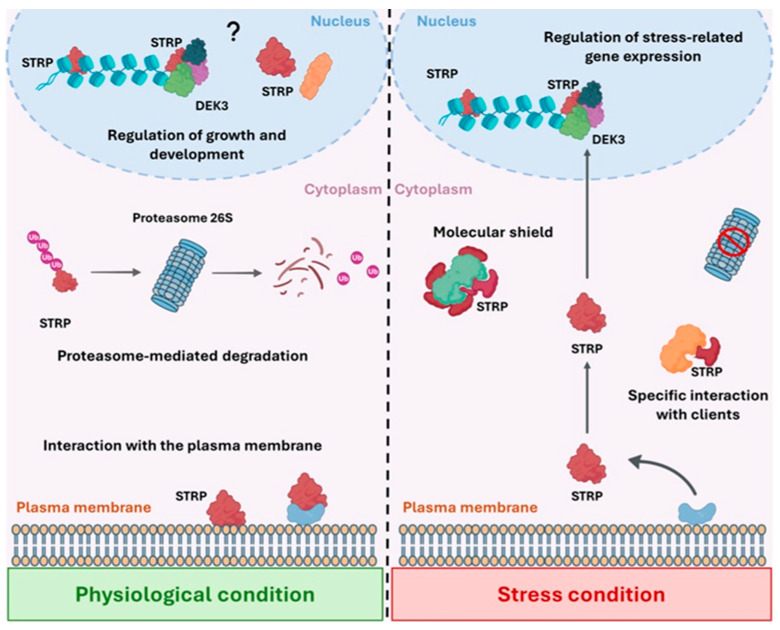
Schematic representation of STRP functional dynamics under physiological and stress conditions. In physiological conditions (**left**), STRP levels are tightly regulated through ubiquitin-mediated proteasomal degradation. A fraction of STRP localizes to the plasma membrane, possibly through interactions with phospholipids or membrane-anchoring proteins, while in the nucleus STRP interacts with histones and the chromatin-associated factor DEK3, suggesting a role in the epigenetic regulation of gene expression involved in controlling growth and development. Upon exposure to abiotic stress (**right**), STRP is no longer targeted for degradation and is released from the plasma membrane, promoting its accumulation in both the cytoplasm and the nucleus, where it exhibits a range of protective functions. STRP can act as a molecular shield, stabilizing partially unfolded or aggregation-prone proteins and preserving protein function during stress exposure. Moreover, the potential ability of STRP to acquire a tertiary structure in a cell context-dependent manner supports its involvement in stress defense mechanisms, also through specific interactions with client proteins. In the nucleus, the STRP interaction with histones and DEK3, and possibly with members of the DEK3 interactome, can contribute to the transcriptional regulation of stress-responsive genes. The functional plasticity of STRP highlights its role as a central mediator of stress adaptation, capable of rapidly modulating its subcellular localization and activity in response to changing environmental conditions.

At the membrane level, STRP may act as a stress sensor and, upon its dissociation, participate in the activation of defense responses (Figure 2). Moreover, the release of STRP from the membrane and its subsequent accumulation in the cytosol and nucleus suggest that the protein primarily exerts its function within these two compartments in response to abiotic stresses. As an IDP, STRP may act as a molecular shield, capable of binding client proteins and DNA, thereby preserving the integrity and function of its targets (Figure 2) [64,76]. Molecular shields are proteins characterized by extensive intrinsic disorder and the absence of a defined three-dimensional structure, playing a critical role in preserving proteome integrity under abiotic stress conditions. Notable examples include LEA proteins and certain small HSPs. These typically lack a stable tertiary structure under non-stress conditions and remain highly soluble even under extreme dehydration, high salinity, or elevated temperatures [63,64]. A hallmark feature of molecular shields is their ability to act as passive stabilizers by forming dynamic and reversible interactions with partially unfolded or aggregation-prone proteins through ATP-independent mechanisms, thereby preventing aggregation via steric hindrance, electrostatic repulsion, and modulation of the local physicochemical environment [63,64]. This functional mode sharply contrasts with classical molecular chaperones, generally well-structured proteins operating through ATP-dependent cycles to facilitate protein folding, unfolding, and refolding [63,64]. Consistent with this distinction, several well-characterized IDPs have been shown to perform protective functions through passive, disorder-based mechanisms, underscoring the evolutionary significance of structural disorder as a plant-specific adaptive strategy in response to environmental stressors [77,78,79]. Therefore, based on the physicochemical and functional properties of STRP, this protein may act as a molecular shield by interacting with diverse molecular partners and contributing to their protection from damage caused by stress-induced alterations of the cellular *milieu*.

Moreover, the physicochemical properties of STRP suggest a potential involvement in promoting biomolecular liquid–liquid phase separation (LLPS), a biophysical process increasingly recognized as essential in plant responses to abiotic stress [80].

Owing to their lack of a stable tertiary structure, IDPs engage in weak, multivalent interactions that favor the assembly of dynamic, membraneless condensates capable of compartmentalizing and regulating key cellular processes during stress [80]. LLPS offers a rapid, tunable, and reversible mechanism to reorganize the intracellular environment in response to fluctuating external factors, facilitating the sequestration, concentration, or spatial redistribution of regulatory components such as mRNAs, transcription factors, and RNA-binding proteins [80].

An expanding repertoire of plant IDPs has been shown to undergo phase separation upon exposure to various abiotic stresses, giving rise to cytoplasmic or nuclear condensates with distinct functional outcomes [80]. Under heat stress, several RNA-binding IDPs assemble into stress granules (SGs) that sequester heat-responsive transcripts and components of the translation machinery, thereby modulating gene expression at the post-transcriptional level. Additional stress conditions, including osmotic and salt stress, also trigger the formation of LLPS-driven condensates involved in transcriptome reprogramming and developmental regulation [80]. While the possible involvement of STRP in LLPS remains speculative and awaits experimental confirmation, it nevertheless represents a promising candidate for future investigations into stress-induced condensate formation in both the nucleus and the cytosol. Specific IDPs can undergo disorder-to-order transitions triggered by stress-induced alterations in the cellular environment. In such cases, IDPs can adopt more structured conformations upon binding to specific molecular partners, such as nucleic acids, lipids, or other proteins [30,31,32]. This conformational shift may modulate their functional activity, enhance binding specificity, or promote the assembly of higher-order complexes, thereby contributing to the reorganization of stress-responsive pathways [31,32]. Given the physicochemical properties of STRP, it is therefore conceivable that, after sensing environmental cues, the protein can adopt secondary structural elements through interaction with specific target proteins, thereby mediating stress-responsive signaling pathways. This mechanism may provide the basis for the interaction between STRP and the chromatin-associated protein DEK3 [19], potentially representing a critical regulatory node through which chromatin structure and gene expression are dynamically modulated in response to environmental stress (Figure 2).

The evidence reviewed here highlights the role of STRP as a multifunctional protein that contributes to mitigating the effects of abiotic stress in *A. thaliana* (Table 1).

Despite recent advances, numerous questions remain unresolved. Future research should focus on characterizing the STRP interactome and its dynamic changes under stress conditions, elucidating the functional significance and regulation of its post-translational modifications, and exploring its interplay with hormonal signaling pathways. By integrating multidisciplinary approaches, deeper insights into the complex mechanisms by which STRP contributes to plant stress adaptation can be gained, thus providing a more comprehensive understanding of plant stress physiology as a whole.

In conclusion, although many aspects of STRP biology remain to be elucidated, its emerging role in abiotic stress responses points to promising prospects in agricultural biotechnology. Exploring its potential in stress tolerance engineering could ultimately contribute to the development of crops with improved resilience and productivity under challenging environmental conditions.

## Figures and Tables

**Table 1:** Milestones of STRP Research.

Year	Discovery	References
2008	First studies on the protective role of STRP in salt stress	[13]
2013	STRP levels increase under temperature stress	[20]
2014	The wheat STRP homolog WCI16 is a LEA-like protein	[28]
2015	STRP is part of the DEK3 interactome	[19]
2020	Protective role in cold stress and involvement in ABA signaling	[14]
2020	STRP is N-terminally acetylated by the NatB complex	[52]
2023	Overexpression of STRP and antioxidant role under salt stress	[15]
2024	STRP is ubiquitinated at multiple Lys residues	[48]

## Data Availability

Data reported from the current study are contained within the article.

## Supplementary Materials

The following supporting information can be downloaded at: https://www.mdpi.com/article/10.3390/plants14192954/s1; Figure S1: phylogenetic analysis of STRP homologs. The tree was generated with PhyloGenes 4.1 (https://phylogenes.arabidopsis.org/tree/PTHR35098, accessed on 8 September 2025) and includes 78 genes from 35 different organisms [28]. Green circles represent speciation nodes, yellow circles duplication nodes, and rhombuses subfamily nodes. The Salt Tolerance-Related Protein is highlighted in red. Figure S2: data plot of *At1g13930* expression levels among different tissues (A) and abiotic stresses (B). Plots were obtained using data from the Public Arabidopsis RNA-Seq Libraries Database PlantRNADB (www.plantrnaDB.com/athrdb, accessed on 20 June 2025) [43].

## Abbreviations

The following abbreviations are used in this manuscript:

STRP	Salt Tolerance-Related Protein
ABA	Abscisic Acid
GRAVY	Grand Average of Hydropathy
NRP1	Nodulin-Related Protein 1
LEA	Late Embryogenesis Abundant
IDP	Intrinsically Disordered Protein
pLDDT	Predicted Local Distance Difference Test
UTR	Untranslated Region
*cis*-nat-siRNA	*cis*-natural antisense small interfering RNA
UPS	Ubiquitin Proteasome System
SCF	SKP1, Cullin, F-box
NatB	N^α^-terminal acetyltransferase B
Nt	N-terminal
HSP	Heat Shock Protein
DEK3	DEK domain-containing protein 3
LLPS	Liquid–Liquid Phase Separation
SG	Stress Granules
