# Supplementary material for: The Emerging Role of the Salt Tolerance-Related Protein in the Abiotic Stress Response of Arabidopsis thaliana
Source: Plants (Basel). 2025 Sep 23;14(19):2954. doi: 10.3390/plants14192954 (PMC12526305; doi:10.3390/plants14192954)
Supplement: Supplementary file 1 [file plants-14-02954-s001.zip › Supplementary Figure S1.pdf]

The Emerging Role of the Salt Tolerance-Related Protein in the Abiotic Stress Response of *Arabidopsis thaliana*

Anna Fiorillo [1], Michela Manai [1], Elisa Falliti [1,2], Sabina Visconti [1] and Lorenzo Camoni [1,*]

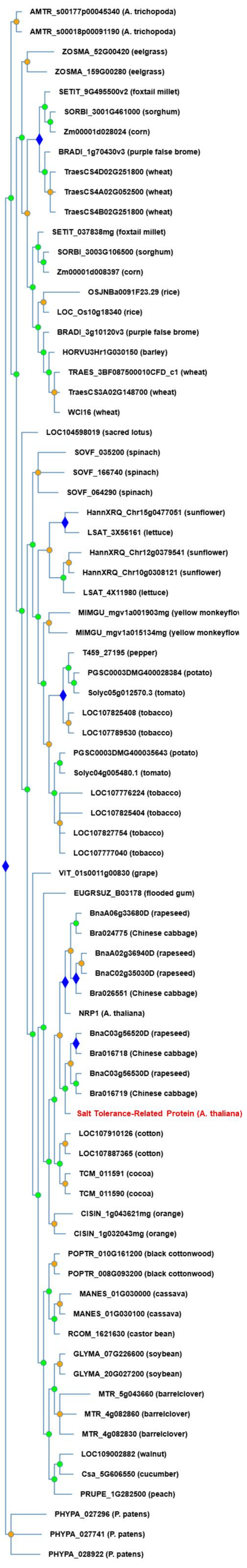

Figure S1: phylogenetic analysis of STRP homologs. The tree was generated with PhyloGenes 4.1 (https://phylogenes.arabidopsis.org/tree/PTHR35098) and includes 78 genes from 35 different organisms [27]. Green circles represent speciation nodes, yellow circles duplication nodes, and rhombuses subfamily nodes. The Salt Tolerance-Related Protein is highlighted in red.
