# Supplementary material for: The Emerging Role of the Salt Tolerance-Related Protein in the Abiotic Stress Response of Arabidopsis thaliana
Source: Plants (Basel). 2025 Sep 23;14(19):2954. doi: 10.3390/plants14192954 (PMC12526305; doi:10.3390/plants14192954)
Supplement: Supplementary file 1 [file plants-14-02954-s001.zip › Supplementary Figure S2.pdf]

The Emerging Role of the Salt Tolerance-Related Protein in the Abiotic Stress Response of *Arabidopsis thaliana*

Anna Fiorillo [1], Michela Manai [1], Elisa Falliti [1,2], Sabina Visconti [1] and Lorenzo Camoni [1,*]

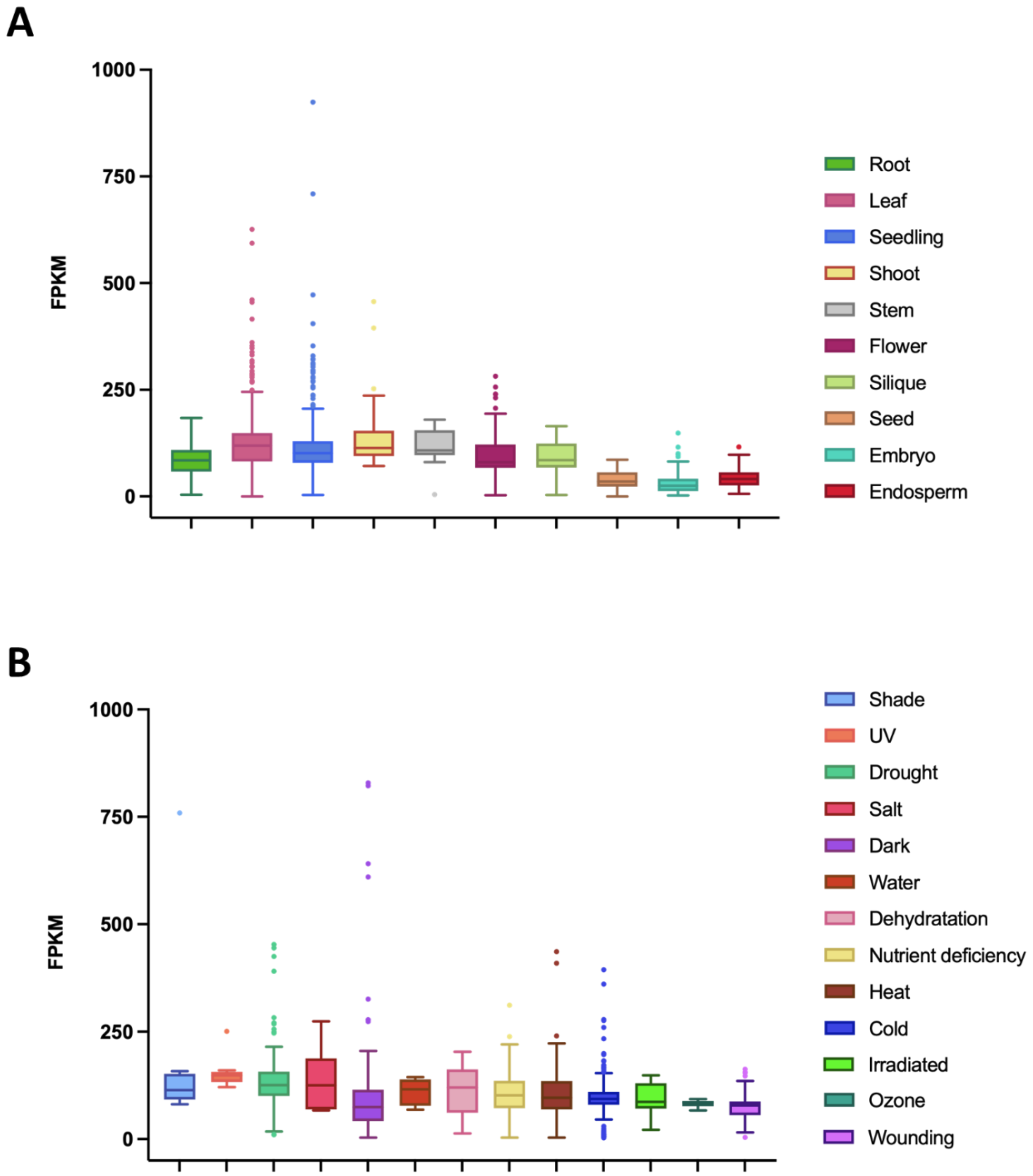

Figure S2: data plot of *At1g13930* expression levels among different tissues (A) and abiotic stresses (B). Plots were obtained using data from the Public Arabidopsis RNA-Seq Libraries Database PlantRNADB (www.plantrnaDB.com/athrdb) [40]. FPKM, Fragments Per Kilobase of transcript per Million mapped reads.
